# Avoidable hospitalizations in Switzerland: a small area analysis on regional variation, density of physicians, hospital supply and rurality

**DOI:** 10.1186/1472-6963-14-289

**Published:** 2014-07-03

**Authors:** Claudia Berlin, André Busato, Thomas Rosemann, Sima Djalali, Maud Maessen

**Affiliations:** 1 Institute of Social and Preventive Medicine, University of Bern, Finkenhubelweg 11, 3012 Bern, Switzerland; 2 Institute of General Practice and Health Services Research, University of Zurich, Pestalozzistrasse 24, 8091 Zurich, Switzerland

**Keywords:** Ambulatory care, Health services, Small area variation analysis, Hospitalization

## Abstract

**Background:**

Avoidable hospitalizations (AH) are hospital admissions for diseases and conditions that could have been prevented by appropriate ambulatory care. We examine regional variation of AH in Switzerland and the factors that determine AH.

**Methods:**

We used hospital service areas, and data from 2008–2010 hospital discharges in Switzerland to examine regional variation in AH. Age and sex standardized AH were the outcome variable, and year of admission, primary care physician density, medical specialist density, rurality, hospital bed density and type of hospital reimbursement system were explanatory variables in our multilevel poisson regression.

**Results:**

Regional differences in AH were as high as 12-fold. Poisson regression showed significant increase of all AH over time. There was a significantly lower rate of all AH in areas with more primary care physicians. Rates increased in areas with more specialists. Rates of all AH also increased where the proportion of residences in rural communities increased. Regional hospital capacity and type of hospital reimbursement did not have significant associations. Inconsistent patterns of significant determinants were found for disease specific analyses.

**Conclusion:**

The identification of regions with high and low AH rates is a starting point for future studies on unwarranted medical procedures, and may help to reduce their incidence. AH have complex multifactorial origins and this study demonstrates that rurality and physician density are relevant determinants. The results are helpful to improve the performance of the outpatient sector with emphasis on local context. Rural and urban differences in health care delivery remain a cause of concern in Switzerland.

## Background

Increasing utilization and rising health care costs threaten the financial sustainability of many western health care systems. Governments and researchers have a growing interest in identifying ineffective and unnecessary health care. Avoidable hospitalizations (AH), also referred to as hospitalizations for ambulatory care sensitive conditions (ACSC) are hospital admissions for diseases and conditions that might have been avoided if better ambulatory care were available [1]. AH are indicators of access and quality of ambulatory care and have been used to monitor health system performance in several countries, including the United States, Canada, Brazil, several European countries, New Zealand and Australia [2-11]. Comparisons between countries that show AH rates for different health care systems may point out strengths and weaknesses of each system.

Structural deficits of care provision such as inequitable access across different societal groups, e.g. urban rural differences, racial and ethnic minorities or different levels of health insurance coverage were identified mainly in the US literature as important predictors of AH’s [12,13]; but AH can also be seen as an indicator of process quality resulting in medical procedures not warranted by effective needs [14]. We are in favor of this process based view as it seems to be more relevant for the Swiss health system which is characterized by high density of supply and generally unlimited access to care [15].

For Switzerland, comprehensive data on AH in different regions and its causal factors are currently unavailable [11]. Switzerland is an interesting country to study AH, because it spends more on health care per capita (5270 USD) than any other country except the United States (8233 USD) and Norway (5388 USD) [16]. Switzerland is also an OECD country with among the highest per capita availability of physicians and nurses and it has one of the world’s highest life expectancies [17].

Health care services in Switzerland are reimbursed by private insurances with compulsory basic health plans that cover a comprehensive catalogue of goods and services for all Swiss inhabitants. There are practically no uninsured patients and limited access to health care is rarely a factor in AH. However, Swiss health care is also characterized by considerable fragmentation due to cantonal autonomy [18] and heterogeneity regarding the distribution of physicians, hospitals and medical facilities, so regional variation of AH rates is to be expected. This project was initiated by the Swiss Federal Office of Public Health (SFOPH) with the goal of documenting regional variation in AH, and identifying relevant determinants of AH rates in Switzerland.

## Methods

### Design of the study

The study is designed as a retrospective analysis of all hospitalizations in Switzerland for the years 2008–2010. In 2002, the OECD initiated the Health Care Quality Indicator Project to measure and compare the quality of health care provision across countries and to develop a set of health care quality indicators [15,19,20]. Within this framework, the OECD uses AH as a measure of quality for prevention and management of chronic diseases in primary care. For comparability reasons, these indicators, including hospitalizations with a principal ICD10 code of asthma, chronic obstructive pulmonary disease (COPD), diabetes complications, congestive heart failure (CHF), and hypertension [19], are used to define AH for this study. Based on OECD eligibility criteria, we included only patients aged 15 and up (15+) and patients not transferred from other hospitals. Detailed ICD codes and additional information on inclusion and exclusion criteria of these conditions as defined by the OECD are given in the Additional file 1.

### Data

We used data from multiple sources. Inpatient care data, including patient demographics, regional data of patient residency, characteristics of hospitalizations (length of stay, type of discharge, referral pathways, health insurance status) and diagnostic and treatment data (ICD10, procedure codes and All Patient Diagnosis Related Groups (APDRG’s)) were extracted from the “Medizinische Statistik der Krankenhäuser”, housed at the Swiss Federal Statistical Office (SFSO) [21]. These data cover all hospitalizations of acute care hospitals in Switzerland. One patient can be hospitalised multiple times (multiple cases). Anonymized unique patient identifiers allow tracking patients across hospitals in case of multiple hospitalizations. Data on structural attributes of acute care hospitals (localization, type, size and specialization of hospitals) are also available from the SFSO (Krankenhausstatistik).

Demographic data at the community level, including age and gender distribution, were available from the SFSO (census data) [22]. In 2010, a new federal population census was introduced by the SFSO (SHAPE project) and the population aged 15+ was used to build the denominator to calculate regional rates of AH for 2010. SHAPE data were also used to directly standardize rates by sex and age groups.

### Geographic unit

Utilization-based health service areas (HSA) of acute care hospitals were the unit of geographic analyses. HSA’s were constructed by analysing discharge data of all acute care hospitals in Switzerland for the period of 2008–2010 [23,24]. We used HSA’s and aggregated zip-code areas of patient residence as the smallest geographic unit (MedStat areas). HSA’s were constructed by cross-tabulating the sum of discharges of every zip-code cluster with all possible hospital regions, and then these regions were merged into an HSA by assigning to the hospital region in which the highest number of patients were treated [25]. Using HSA’s has the advantage of describing where patients actually receive care, without regard to cantonal or other administrative borders [21,23,24,26,27]. This approach is well established and has become an indispensable source of information for current US healthcare reformers [28].

In 2008, the SFSO modified the concept of MedStat areas to make them compatible with other geographic classification systems. However, these changes were not equally implemented in the data collection procedures of all Swiss cantons during the course of the study. In consequence, geographic classification of hospitals and of patient residence was inconsistent in some cantons. Data from cantons Appenzell Innerrhoden, Appenzell Ausserrhoden, Schaffhausen, St. Gallen, Thurgau and Zurich were therefore discarded from the small area analysis.

### Statistical procedures

All hospitalizations corresponding to the list of AH published by the OECD were included [15]. Statistical analysis of the data was performed in two steps. The first included a descriptive nationwide analysis of AH; the second step identified determinants of regional differences of AH-rates in cantons with eligible data. Descriptive procedures documented overall rates, demographic characteristics, comorbid conditions of patients (Charlson index [29]), length of stay, APDRG cost-weights, and inpatient mortality of all AH in Switzerland. APDRG cost weights that accounted for outliers of length of stay were calculated according to version 6.0 of the specifications of APDRG-Suisse [30].

Regional rates of AH were calculated at the level of zip-code clusters; the number of AH admittances in the numerator and the total regional population aged 15+ were the denominator. Direct standardization of rates by sex and age was performed at the level of zip-code clusters and used the total 15+ Swiss population of 2010 as the reference.

For geographic analyses we summarized the data at two levels (utilization-based health service areas [n = 59] and aggregated zip-code clusters [n = 436]) and developed statistical models to explore the relationship between rates of AH and characteristics of regional supply of medical care. For each zip code cluster, we determined the density of primary care physicians and of specialists in own practice (physicians per 10000 population). Physician groups were defined based on definitions established by the Swiss Medical association [31]. We also calculated the proportion of the population living in rural communities [32]. At the level of HSA’s we calculated the number of acute care hospital beds per 10000 people as a measure of regional hospital supply.

We used a multilevel poisson regression model with the natural logarithm of the age and sex standardized number of AH as the outcome. We used the log number of the population of zip-code clusters as a fixed offset term in the regression equation. We added information on regional supply of ambulatory care and of population characteristics at the level of zip-code clusters, and added predictors at the level of HSA to estimate effects related to hospital supply. The final set of explanatory variables was obtained after a series of preliminary analyses that explored bivariate associations between the outcome and various measures of physician’s supply, including full time equivalents, and other methods to classify patient residency geographically. We eventually included explanatory variables which explained the largest variability of AH admissions.

These are the explanatory variables included in the final model:

Level 1 (436 zip code areas)

– Year (2008, 2009, 2010)

– Number of primary care physicians per 10000 population

– Number of specialists per 10000 population (all medical specialists with office based practice)

– Proportion of the population living in rural areas

– Type of hospital reimbursement system (APDRG vs. other systems)

– Level 2 (59 utilization based health service areas)

– Number of hospital beds per 10000 population

We added random intercepts at the level of HSA’s and zip-code clusters to allow for unexplained variation around the respective means. We used the same model to analyse effects associated with the overall rate, and for condition specific rates of AH’s. In order to explore linear relationships between continuous explanatory variables and AH, we additionally defined a second model and replaced the continuous data with quintiles of the respective variables. Differences to the first quintile were documented as incidence risk ratios. SAS 9.3 (proc GLIMMIX) was used for multilevel modeling and ArcGis 10 to create maps, the level of significance was set to p < 0.05 throughout the study.

## Results

### Characteristics of avoidable hospitalizations

For 2008–2010, 3470812 hospitalizations of patients were documented in the discharge data of the “Medizinische Statistik der Krankenhäuser”. Of these, 92804 hospitalizations fulfilled the OECD inclusion criteria and had an ICD10 diagnose that corresponded with a AH. The overall rate of AH for the 15+ population during this period was 467 hospitalizations per 100000. The respective rates were 455 for women and 483 for men. From 2008 to 2010, we observed an increase of 2.7%. Annual rates for 2008–2010 were 463, 467 and 476 per 100000. In 2010, AH accounted for 3.1% of all hospital stays (n = 31805) and generated 180–200 Mio CHF of direct hospital costs (depending on annual cantonal APDRG base rates). The proportion of patients with additional health insurance in the study population was 15.2% — slightly below the Swiss average of 16.6% of all hospitalized patients. 74.3% of the AH were classified as emergencies, i.e. with a need for treatment within 12 hours, and 11.8% of patients were re-hospitalized within 3 months. On average, patients with a rehospitalization had 2.7 hospitalizations during the study period (2.3 for hypertension, 2.6 for Asthma, CHF and Diabetes and 3.0 for COPD). Inpatient mortality of AH was 5.1%. Characteristics of disease specific AH are given in Table 1.

**Table 1:** Major characteristics of avoidable hospitalizations across ICD10 groups in Switzerland 2008-2010

**ICD10 Group**	Rate^*^	**Avg. age of patients**	**Avg. length of stay (days)**	**Emergency admissions**	**In-hospital deaths**	**3-month Rehospitalization**^**§**^
Asthma	18.6	54.1	6.8	84.0%	0.5%	5.6%
CHF^†^	211.0	78.8	12.2	78.1%	9.0%	11.5%
COPD^‡^	110.0	71.3	11.3	73.7%	3.6%	14.9%
Diabetes	70.7	62.6	12.2	63.7%	1.0%	7.5%
Hypertension	58.3	70.1	6.3	71.4%	0.4%	3.5%
All Avoidable hospitalizations	468.6	72.5	11.0	74.3%	5.1%	10.4%

^*^Rate per 100000 total population.

^†^Congestive heart failure.

^‡^Chronic obstructive pulmonary disease.

^§^Percentage of patient rehospitalized within 3 months after the initial hospitalization.

### Hospital characteristics

We used the specification of the SFSO to categorize hospitals into five hospital groups based on annual number of all hospitalizations. Characteristics of AH across these groups are given in Table 2. It is important to note the differences between low-volume clinics, usually located in peripheral areas, and high volume clinics, located in urban areas. There was almost twice the proportion of AH in low-volume clinics (4% vs. 2%); average length of stay was six days longer; patients were older; and, in-hospital mortality was higher. In high volume clinics, APDRG cost-weights per case were almost double those of low volume clinics.

**Table 2:** Characteristics of avoidable hospitalizations across different acute care hospital groups

	**% avoidable hospitalizations**^**† **^**(%)**	**Patient age (mean)**	**Length of stay (days)**	**Charlson index (mean)**	**Cost weights**^**‡ **^**(mean)**	**3-month Rehospitalization (%)**	**In-hospital Mortality (%)**
Hospital group^*^							
Centrally provided treatments 1 (>30′000 admittances/year)	2.1	68.2	10.5	0.23	2.33	9.8%	4.1%
Centrally provided treatments 2 (9′000-30′000 admittances/year)	2.9	73.0	10.6	0.29	1.12	9.8%	5.5%
Basic care 1 (6′000-9′000 admittances/year)	3.0	70.8	10.0	0.31	1.14	9.4%	5.1%
Basic care 2 (3′000-6′000 admittances/year)	2.2	70.4	10.8	0.21	1.29	10.0%	4.2%
Basic care 3 (<3000 admittances/year)	4.0	74.0	16.8	0.20	1.26	13.2%	6.3%

^*^Based on definitions of the Swiss Federal Statistical office.

^†^Proportion of avoidable hospitalizations among all hospitalizations.

^‡^Average APDRG cost weights per avoidable hospitalization.

### Regional differences

**Figure 1:**
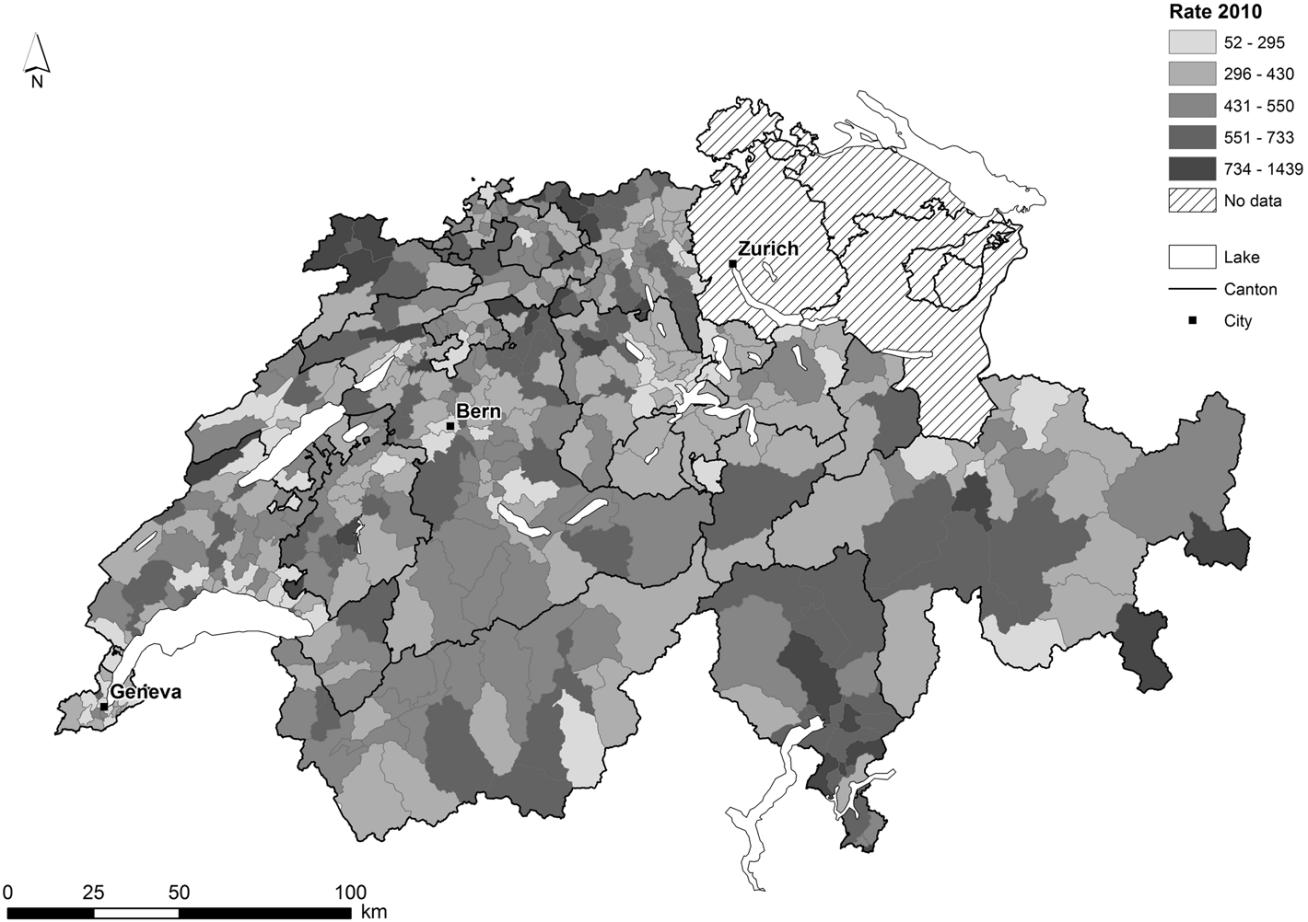
Age and sex standardized rates per 100000 inhabitants of avoidable hospitalizations for 2010 of 436 zip-code clusters.

**Figure 2:**
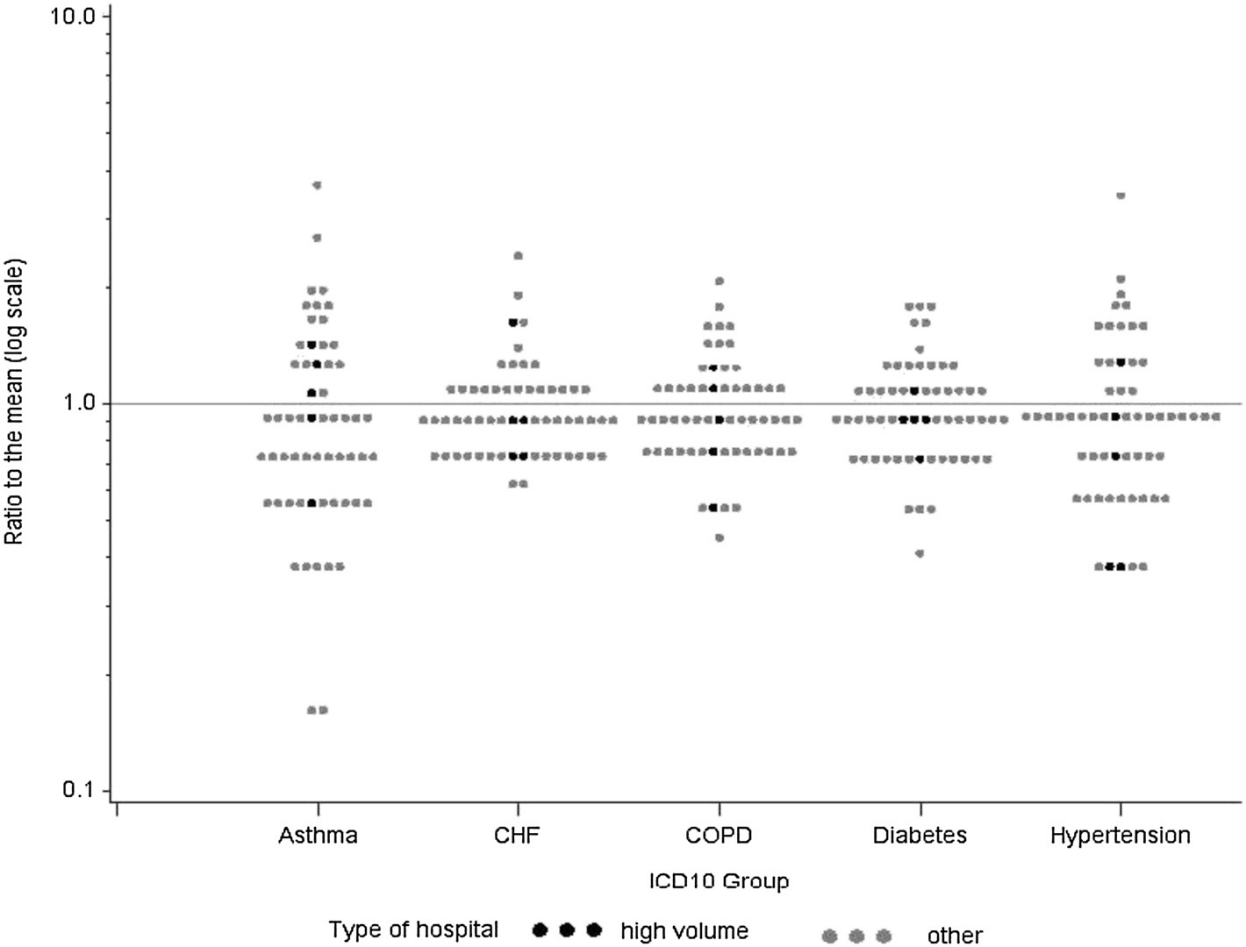
Regional variation (59 HSA’s) of disease specific avoidable hospitalization rates^*^. *Each dot represents a HSA, rates are plotted as a ratio to the mean on a log scale. black dots denote HSA’s with at least on high volume clinic.

The 59 HSA’s included in the study covered 87.0% of the area of Switzerland and 69.2% of the Swiss population in 2010. Key characteristics of these areas are given in Table 3. Depending on year, we observed up to 12-fold regional differences of age and sex standardized rates of AH across HSA’s. As an example, geographic patterns are documented for 2010 in Figure 1. The three year averages of AH rates of HSA’s showed only a 3.6-fold regional variation (range: 274–982 admittances per 100000 population). Rate ratios to the mean of the three year averages were used to document the regional variation of AH across disease groups (Figure 2). This data implies particularly high levels of variation for asthma and hypertension and less variation for congestive heart failure, COPD and diabetes.

**Table 3:** Regional variation, characteristics of 59 health service areas (averages of 2008–2010)

**Characteristic**	**Average**	**Min**	**Max**	**HILO ratio**^**‡**^
Population size	94301	1614	507789	314.6
Population density^*^	303.4	26.4	1863.0	70.6
Number of hospitals	2.4	1	12	12.0
Hospital beds per 10000	41.1	13.5	414.0	30.7
Primary care physicians per 10000	6.5	1.9	10.9	5.7
Specialists per 10000	2.1	0.0	6.2	?
-Rate of avoidable hospitalizations^†^				
- Asthma	21.5	2.2	79.5	36.1
- congestive heart failure	236.2	146.0	569.2	3.9
- COPD	122.0	55.4	252.9	4.6
- Diabetes	74.1	30.4	136.3	4.5
- Hypertension	59.9	18.4	207.0	11.3
- all ICD10 groups	495.2	274.5	982.4	3.6

^*^Population per km^2^ surface below 2000 meters altitude.

^†^Age and sex standardized rate per 100000 population.

^‡^Ratio of highest vs. lowest values.

The results of multilevel modelling are summarized in Table 4. The data show a significant increase in all AH over time, and significantly lower rates for all AH areas with more primary care physicians. Rates were increased in areas with more specialists, and also increased rates in areas with a higher proportion of rural residents. There was an insignificant association of all AH for regional hospital capacity (hospital beds per 10000 population) and for type of hospital reimbursement (APDRG vs. other). Quintiles of supply and population data show a 0.91 times lower incidence of AH in areas with more than 8.1 primary care physicians per 10000 compared to areas with less than 3.8 physicians (p < 0.05); there was a 1.15 times higher incidence in areas with more than 4.2 specialists in comparison to areas with zero specialists (p < 0.05) and a 1.12 times greater incidence in areas where more than 42% of the population lived in rural communities (reference: areas with zero rural residents, p < 0.05) (Table 4).

**Table 4:** Incidence rate ratios of health system characteristics associated with avoidable hospitalizations

**Characteristic**	**All avoidable hospitalizations**	**Asthma**	**CHF**	**COPD**	**Diabetes**	**Hypertension**
Intercept	0.005^*^	0.000^*^	0.002^*^	0.001^*^	0.001^*^	0.001^*^
Year 2008^†^	-	-	-	-	-	-
Year 2009	1.155^*^	0.979	0.923^*^	1.011	0.954	1.066^*^
Year 2010	1.168^*^	1.105^*^	0.940^*^	1.042^*^	0.904^*^	1.016
Primary care physicians per 10000	0.986^*^	0.989	0.998	1.006	1.015^*^	1.003
Q1^‡^	-	-	-	-	-	-
Q2	1.033	0.827^*^	1.060	1.043	1.174^*^	1.064
Q3	1.028	0.819^*^	0.996	1.049	1.152^*^	1.043
Q4	1.043	0.769^*^	1.021	1.046	1.132^*^	1.065
Q5	0.905^*^	0.883	1.002	1.029	1.189^*^	1.020
Specialists per 10000	1.017*	1.048*	1.017*	1.017*	1.019*	1.013
Q1^‡^	-	-	-	-	-	-
Q2	1.019	1.162	0.961	0.996	1.073	1.018
Q3	1.066^*^	1.044	1.008	1.031	1.126^*^	0.985
Q4	1.067^*^	1.262^*^	1.040	1.101^*^	1.063	1.005
Q5	1.152^*^	1.330^*^	1.132^*^	1.125^*^	1.162^*^	1.131
Proportion of residents in rural communities	1.113*	0.799	1.207*	1.061	0.981	0.888
Q1^‡^	-	-	-	-	-	-
Q2	0.987	0.918	1.023	0.895^*^	0.999	0.887
Q3	1.038	0.842	1.044	1.148^*^	0.990	0.967
Q4	1.037	0.771^*^	1.047	1.006	1.055	0.972
Q5	1.115^*^	0.859	1.171^*^	1.022	1.009	0.897
Hospital beds per 10000	1.000	1.001	0.999	1.001	1.000	0.998
Q1^‡^	-	-	-	-	-	-
Q2	1.008	1.144	1.040	0.959	1.134	0.918
Q3	1.023	1.076	1.027	1.069	1.141	0.804^*^
Q4	1.026	1.023	1.032	1.073	1.141	0.838^*^
Q5	1.024	1.062	1.001	1.069	1.070	0.877
Type of hospital reimbursement	0.949	1.414^*^	1.005	0.909	0.932	0.662^*^

^*^Significant incidence risk ratio, p < 0.05.

^†^Year 2008 as the reference.

^‡^Incidence risk ratio from a model with annual quintiles of continuous explanatory variables with the first quintile as the reference, quintiles are varying across years therefore no threshold levels are given in the table.

Inconsistent patterns of significant incidence ratios across different disease groups were observed for regional care supply. For asthma, a higher number of primary care physicians was partially associated with fewer AH, but increased rates of AH were observed for diabetic patients in areas with more primary care physicians. We observed more consistent significant associations of AH with regional specialist supply; higher supply was associated with higher rates irrespective of disease group. Inconsistent and partially non-linear relationships across disease groups were also seen for significant associations of AH with more rural populations (Table 4).

## Discussion

Our study highlights up to 12-fold regional differences of AH over a period of three years. Research on medical practice variation shows that regional differences of this extent are mainly associated with the medical care system including provider factors and less to regional variation in the incidence of the underlying disease categories [8,9,33]. Comparative data across countries for avoidable hospitalizations are available from the OECD for Asthma, COPD and uncontrolled diabetes [17]. These data show low overall rates of avoidable hospitalizations for all three conditions in Switzerland implying a high quality of primary care. However, patterns of variation observed in our data point to difficulties of the Swiss health system to provide effective and equitable medical care to all societal groups.

Theoretically, hospital admissions for ACSC can be prevented by effective ambulatory care, irrespective of disease prevalence. However in practice, even the best ambulatory care may not be able to prevent these hospitalizations. Some factors are beyond the scope of ambulatory care providers, for example: different propensity of patients to seek care; advanced stage and complexity of some conditions; lack of compliance with preventive measures; financial constraints; and, poor access to transportation. To some degree, regional differences of AH rates will continue to reflect differences in prevalence of the underlying disease. However, studies which took regional variation of health status into account still found an independent association between AH and care supply [34,35]. It is therefore unlikely that adjacent geographical areas have a high enough difference in prevalence to produce up to 12-fold regional differences of AH. Our results also showed that, irrespective of condition, the majority of AH were categorized as emergencies. These findings may raise questions about how avoidable these hospitalizations are, but they may also suggest inappropriate use of costly emergency services and indicate non-optimal quality and efficacy of outpatient care in Switzerland. Our data allow no further analysis of potential causes of emergency admissions as the data obtained from the Federal Statistical Office contain no variables on type of physicians referring patients to a hospital.

### Characteristics of supply

Regional variation in the data suggest distinct differences between the decisions of primary care physicians and specialists to admit patients to hospitals, even demographic characteristics of patients and higher rates of AH in predominantly rural populations are both accounted for. An explanation of the patterns would require further comparison of physician practice styles. Risk ratios show that patients living in areas with a high density of primary care physicians are less likely to be admitted to hospitals for conditions that can be treated in ambulatory care. But it is important to note that we observed mostly non-linear relationships between AH and primary care supply. Our results show that AH can only be reduced in areas with very high density of primary care physicians. We also found differences across major disease groups indicating that primary care physicians may have varying ability to treat health problems and to prevent exacerbations, depending on the conditions they treat.

Our general findings are consistent with multiple studies performed in many countries, with a variety of health care delivery systems [35-37]. The data highlight the importance of primary care as an effective first-contact access to health care irrespective of the characteristics of the health system in which care takes place.

In contrast to the data on primary care physicians, we observed a consistent pattern of higher risks of AH in areas with a high density of specialists who work in own practices. Specialist practices in Switzerland are almost exclusively located near hospitals (not shown). We cannot confirm other research that established an association between AH and regional hospital beds supply [38]. Our findings suggest that primary care physicians and specialists have different priorities when they refer patients to hospitals, irrespective of regional hospital capacity.

It may be that specialists are treating more patients with more severe conditions which are more likely to require hospitalization. Our analyses, however, used age and sex standardized population-based rates on a small area scale as an outcome, and it is unlikely that populations in areas with high specialist density are characterized by a higher burden of disease. Specialists working in own practice are often affiliated with local hospitals and, to some degree, hospital admissions may be influenced by financial incentives [39].

We observed distinct differences between patient characteristics and outcome indicators for high- and low volume clinics. AH in low-volume clinics were characterized by older patients with fewer comorbid conditions, higher 3-month rehospitalization rates, and higher in-patient mortality. These results appear contradictory and raise questions about the validity of diagnostic data; it is unlikely that older patients have lower burden of disease and fewer comorbid conditions. We speculate that this may be explained by different procedures for coding diagnoses across hospitals. Larger hospitals usually have dedicated staff for his task, while coding in smaller hospitals is normally done by clinicians.

Hospital data also show higher proportions of AH and considerable longer hospital stays in low volume clinics. Consistent with our geographic analysis of rurality, and with other research [40], these results suggest potential effects scarcity of outpatient resources in proximity to low-volume clinics. However, we cannot exclude in this setting that supply sensitive effects of such hospitals are also promoting AH in order to legitimate the viability of low-volume hospitals. Due to lack of data, we cannot discriminate between the two mechanisms.

### Patient characteristics

Our data showed more AH in men and confirmed gender associated differences documented in other research [20,41]. Our evidence on the socio-economic status of patients was in conflict with other research [37,42]. Some characteristics of Swiss health care are therefore important to note. All Swiss residents are required to purchase compulsory health insurance, covering a comprehensive catalogue of goods and services. Residents may also have supplementary health insurance contracts that typically provide superior levels of accommodation, give more choice of in-hospital physicians in hospitals and may provide cash benefits for sickness absence [18]. Data from the 2007 Swiss health survey shows that supplementary health insurance is typically purchased by those with high income [43]. Type of insurance coverage is thus a good proxy for the socio-economic status of patients. Our data on health insurance status of patients hospitalized for ACSC show 1.4% less patients with supplementary coverage compared to the overall Swiss average of 16.6% of patients with supplementary coverage. The results do not support the evidence that AH is influenced by a socioeconomic gradient in the setting of Swiss health care.

As in previous research [40,44], regional variation in our data indicates positive associations between AH and rurality of patient residence. Appropriate allocation of resources in rural areas is of concern to health care planners. However, we cannot distinguish between the effects of limited access to care and patient level factors that have been observed among rural residents in previous research, such as lower propensity to seek care [7]. More data is required to explain this regional variation within Swiss health care, and until it is collected and analyzed, specific policy recommendations cannot be made.

### Strengths and limitations

Although we used hierarchical mixed models with random effects at the level of HSA’s and zip-code clusters, which should address some concerns about unmeasured variables, it is important to recognize that health care delivery in the out-patient sector is highly complex. Statistical modelling is difficult because factors like physician behaviour, perceptions of quality of the interaction between patients and physicians, social status of patients and cultural norms including differential propensity among subpopulations to seek care are not fully understood [45]. The search for determinants is further complicated by a scarcity of data that measures the impact of different forms of care delivery on patient health at the health system level.

We were also limited by the lack of ambulatory care sensitive conditions specifically validated as indicators of quality of care for Switzerland. When using administrative data it is not always possible to directly discriminate between AH and necessary hospitalizations. A major limitation is that we have no knowledge about the patient’s history of disease before the admission to the hospital: We don’t know whether the patient’s disease or symptoms of the disease were diagnosed, appropriate treated and monitored by ambulatory care before [1]. So we cannot determine with certainty that the hospitalization was clinically preventable or necessary. Although this misclassification on clinical level is unfortunate, we believe that the AH selection of the OECD can still be useful as a health indicator from a health service perspective.

The concept of avoidable hospitalizations is based on specific diagnoses that should not be treated in hospitals. Database information allows therefore only indirect identification of avoidable hospitalizations. We used the criteria of the OECD initiated Health Care Quality Indicator Project as the case definition of AH, but clinical criteria and appropriateness of hospitalization remains subject of constant debate. It will continue to be, as long as the concept of AH is not validated by patient-level outcomes. The concept of AH does not take into account the potential benefits for patients of a theoretically avoidable hospital stay. But as long as valid data regarding the outcome of hospitalizations on patient health remain unavailable, AH is currently the best approach for estimating the appropriateness of care.

We used administrative hospital data not specifically designed for this type of research, which caused a number of problems. Our results indicate that completeness and accuracy of coding diagnoses may differ between hospitals and that accuracy of geographic data of hospital location and patient residence were compromised in some cantons. This forced us to exclude data from six cantons for small area analysis. These difficulties directly reflect effects of federalism and cantonal autonomy in Swiss health care and point to an urgent need to improve the quality of nationwide health data collection.

Finally, we used age and sex standardized rates of AH for the whole population as the outcome in this study. This outcome may underestimate the overall burden of ACSC, and rates adjusted to the underlying disease prevalence should be used instead [46]. Unfortunately, small area data for disease prevalence are not available, and an expensive data collection processes would be required to obtain such data. However regional differences of rates of AH can also be interpreted as differential ability of a health system to meet region-specific burdens of ACSC; a prevalence adjusted analysis would obliterate such differences.

The major strength of this study is its nationwide approach; it gives insight into AH rates that transcend cantonal administrative boundaries. It proves that small area analysis in a complex setting is feasible, and has opened the door to further monitoring of regional variations of health interventions, which will support evidence-based policy making in Swiss health care. Another strength is our use of OECD definitions for AH and reported disease specific characteristics. This well established approach improves the generalizability of our data and allows comparative analyses across multiple health systems [15].

## Conclusions

We identified significant determinants of AH in the Swiss health system that are important for health care planning. We uncovered disease specific characteristics of AH, indicating that disease specific health policy may be effective. However, we are limited by the quality of the data we used; more valid data must be collected by hospitals and ambulatory care providers. Our results may be used to improve the performance of the outpatient sector, particularly in local regional contexts. Rural and urban differences remain a cause of concern. Future research should assess specific physician characteristics that contribute to AH, with the goal of reducing the number of unnecessary procedures.

## Abbreviations

AH: Avoidable hospitalizations; ACSC: Ambulatory care sensitive conditions; COPD: Chronic obstructive pulmonary disease; CHF: Congestive heart failure; SFOPH: Swiss Federal Office of Public Health; OECD: Organization for economic co-operation and development; SHAPE: System of household and personal statistics; HSA: Health service areas; ICD10: The International Classification of Diseases version 10; APDRG: All patient diagnosis related groups; CHF: Swiss Franc.

## Competing interests

All authors declare that they have no competing interests.

## Authors’ contributions

CB analysed the validity of the geographic data, produced the geographical map and interpreted the results. AB, deceased on 12^th^ November 2013, designed the original study project and performed the data analyses. MM helped drafting the manuscript and interpreting and prioritising the results. All authors contributed to revising the manuscript and approved the final manuscript.

## Pre-publication history

The pre-publication history for this paper can be accessed here:

http://www.biomedcentral.com/1472-6963/14/289/prepub

## Supplementary Material

Additional file 1 These tables represent the in- and exclusion criteria for avoidable hospitalizations based of on the criteria from the OECD Health Care Quality Indicator Project. Click here for file
