# Supplementary material for: Avoidable hospitalizations in Switzerland: a small area analysis on regional variation, density of physicians, hospital supply and rurality
Source: BMC Health Serv Res. 2014 Jul 3;14:289. doi: 10.1186/1472-6963-14-289 (PMC4091658; doi:10.1186/1472-6963-14-289)
Supplement: Additional file 1 — These tables represent the in- and exclusion criteria for avoidable hospitalizations based of on the criteria from the OECD Health Care Quality Indicator Project. [file 1472-6963-14-289-S1.docx]

## Additional file

These tables represent the in- and exclusion criteria for avoidable hospitalizations based of on the criteria from the OECD Health Care Quality Indicator Project.

| **Asthma admission rate** | |
| --- | --- |
| **Numerator:** All non-maternal hospital discharges (age 15+) with a principal diagnosis code of asthma in a specified year. | |
|  | |
| Asthma diagnosis codes: | |
| **ICD-9-CM** | **ICD-10-WHO** |
| 49300 Ext Asthma W/O Stat Ash | J450 Predominantly allergic asthma |
| 49301 Ext Asthma W Status Ash | J451 Nonallergic asthma |
| 49302 Ext Asthma W Acute Exac Oct00- | J458 Mixed asthma |
| 49310 Int Asthma W/O Stat Asth | J459 Asthma, unspecified |
| 49311 Int Asthma W Status Asth | J46 Status asthmaticus |
| 49312 Int Asthma W Acute Exac Oct00- |  |
| 49320 Ch Ob Asth W/O Stat Asth |  |
| 49321 Ch Ob Asthma W Stat Asth |  |
| 49322 Ch Obs Asth W Acute Exac Oct00- |  |
| 49381 Exercse Ind Bronchospasm Oct03- |  |
| 49382 Cough Variant Asthma Oct03- |  |
| 49390 Asthma W/O Status Asthm |  |
| 49391 Asthma W Status Asthmat |  |
| 49392 Asthma W Acute Exacerbtn Oct00- |  |
| Exclude cases: | |
| - transferring from another institution | |
| - pregnancy, childbirth, and puerperium | |
| - newborn and other neonates | |
| - with any diagnosis code of cystic fibrosis and anomalies of the respiratory system | |
| - same day/day only admissions (admissions with a length of stay less than 24 hours. In those countries where a timestamp of admission or discharge is not available cases with a length of stay of 0 days shall be excluded. | |
|  | |
| Exclude diagnostic codes cystic fibrosis and anomalies of the respiratory system: | |
|  |  |
| **ICD-9-CM** | **ICD-10-WHO** |
| 27700 Cystic Fibros W/O Ileus | E840 Cystic fibrosis with pulmonary manifestations |
| 27701 Cystic Fibros W Ileus | E841 Cystic fibrosis with intestinal manifestations |
| 27702 Cystic Fibros W Pul Man | E848 Cystic fibrosis with other manifestations |
| 27703 Cystic Fibrosis W Gi Man | E849 Cystic fibrosis, unspecified |
| 27709 Cystic Fibrosis Nec | P27.0 Wilson-Mikity syndrome |
| 74721 Anomalies Of Aortic Arch | P27.1 Bronchopulmonary dysplasia originating in the perinatal period |
| 7483 Laryngotrach Anomaly Nec | P27.8 Other chronic respiratory diseases originating in the perinatal period |
| 7484 Congenital Cystic Lung | P27.9 Unspecified chronic resp disease originating in the perinatal period |
| 7485 Agenesis Of Lung | Q25.4 Other congenital malformations of aorta |
| 74860 Lung Anomaly Nos | Q31.1 Congenital subglottic stenosis |
| 74861 Congen Bronchiectasis | Q31.2 Laryngeal hypoplasia |
| 74869 Lung Anomaly Nec | Q31.3 Laryngocele |
| 7488 Respiratory Anomaly Nec | Q31.5 Congenital laryngomalacia |
| 7489 Respiratory Anomaly Nos | Q31.8 Other congenital malformations of larynx |
| 7503 Cong Esoph Fistula/Atres | Q31.9 Congenital malformation of larynx, unspecified |
| 7593 Situs Inversus | Q32.0 Congenital tracheomalacia |
| 7707 Perinatal Chr Resp Dis | Q32.1 Other congenital malformations of trachea |
|  | Q32.2 Congenital bronchomalacia |
|  | Q32.3 Congenital stenosis of bronchus |
|  | Q32.4 Other congenital malformations of bronchus |
|  | Q33.0 Congenital cystic lung |
|  | Q33.1 Accessory lobe of lung |
|  | Q33.2 Sequestration of lung |
|  | Q33.3 Agenesis of lung |
|  | Q33.4 Congenital bronchiectasis |
|  | Q33.5 Ectopic tissue in lung |
|  | Q33.6 Hypoplasia and dysplasia of lung |
|  | Q33.8 Other congenital malformations of lung |
|  | Q33.9 Congenital malformation of lung, unspecified |
|  | Q34.0 Anomaly of pleura |
|  | Q34.1 Congenital cyst of mediastinum |
|  | Q34.8 Other specified congenital malformations of respiratory system |
|  | Q34.9 Congenital malformation of respiratory system, unspecified |
|  | Q39.0 Atresia of oesophagus without fistula |
|  | Q39.1 Atresia of oesophagus with tracheo-oesophageal fistula |
|  | Q39.2 Congenital tracheo-oesophageal fistula without atresia |
|  | Q39.3 Congenital stenosis and stricture of oesophagus |
|  | Q39.4 Oesophageal web |
|  | Q39.8 Other congenital malformations of oesophagus |
|  | Q89.3 Situs inversus |
|  | |
|  | |
| **Denominator:** 100,000 Population (age 15+ years). | |

| **COPD admission rate** | |
| --- | --- |
| **Numerator:** All non-maternal hospital discharges (age 15+) with a principal diagnosis code for Chronic Obstructive Pulmonary Disease (COPD) in a specified year. | |
|  | |
| COPD diagnosis codes: | |
|  | |
| **ICD-9-CM** | **ICD-10-WHO** |
| 490 BRONCHITIS NOS* | J40 Bronchitis* |
| 4660 AC BRONCHITIS* | J410 Simple chronic bronchitis |
| 4910 Simple Chr Bronchitis | J411 Mucopurulent chronic bronchitis |
| 4911 Mucopurul Chr Bronchitis | J418 Mixed simple and mucopurulent chronic bronchitis |
| 49120 Obs Chr Brnc W/O Act Exa | J42 Unspecified chronic bronchitis |
| 49121 Obs Chr Brnc W Act Exa | J430 MacLeod's syndrome |
| 4918 Chronic Bronchitis Nec | J431 Panlobular emphysema |
| 4919 Chronic Bronchitis Nos | J432 Centrilobular emphysema |
| 4920 Emphysematous Bleb | J438 Other emphysema |
| 4928 Emphysema Nec | J439 Emphysema, unspecified |
| 494 Bronchiectasis Oct00- | J440 COPD with acute lower respiratory infection |
| 4940 Bronchiectas W/O Ac Exac Oct00- | J441 COPD with acute exacerbation, unspecified |
| 4941 Bronchiectasis W Ac Exac Oct00- | J448 Other specified chronic obstructive pulmonary disease |
| 496 Chr Airway Obstruct Nec | J449 Chronic obstructive pulmonary disease, unspecified |
|  | J47 Bronchiectasis |
| **Qualifies only if accompanied by secondary diagnosis of 491.xx, 492.x, 494.x or 496 (i.e., any other code on this list).* | **Qualifies only if accompanied by secondary diagnosis of J41, J43, J44, J47* |
|  |  |
|  | |
|  | |
| Exclude cases: | |
| - transferring from another institution | |
| - pregnancy, childbirth, and puerperium | |
| - newborn and other neonates) | |
| - same day/day only admissions (admissions with a length of stay less than 24 hours. In those countries where a timestamp of admission or discharge is not available cases with a length of stay of 0 days shall be excluded. | |
|  | |
| **Denominator:** 100,000 Population (age 15+ years). | |

| **Diabetes lower extremity amputation rate** | |
| --- | --- |
| **Numerator:** All non-maternal discharges (age 15+) with procedure code for lower extremity amputation in any field and diagnosis code of diabetes in any field in a specified year. | |
|  | |
| Diabetes lower extremity amputation diagnostic codes: | |
| **ICD-9-CM** | **ICD-10-WHO** |
| *Procedure codes for lower-extremity amputation* | *Procedure codes for lower-extremity amputation* |
|  |  |
| 8410 Lower Limb Amputat Nos | Not specified |
| 8411 Toe Amputation |  |
| 8412 Amputation Through Foot |  |
| 8413 Disarticulation Of Ankle |  |
| 8414 Amputat Through Malleoli |  |
| 8415 Below Knee Amputat Nec |  |
| 8416 Disarticulation Of Knee |  |
| 8417 Above Knee Amputation |  |
| 8418 Disarticulation Of Hip |  |
| 8419 Hindquarter Amputation |  |
|  |  |
| *Diagnosis Codes For Diabetes:* | *Diagnosis codes* *for diabetes:* |
|  |  |
| 25000 Dmii Wo Cmp Nt St Uncntr | E10.0 Insulin-dependent diabetes mellitus with coma |
| 25001 Dmi Wo Cmp Nt St Uncntrl | E10.1 Insulin-dependent diabetes mellitus with ketoacidosis |
| 25002 Dmii Wo Cmp Uncntrld | E10.2 Insulin-dependent diabetes mellitus with renal complications |
| 25003 Dmi Wo Cmp Uncntrld | E10.3 Insulin-dependent diabetes mellitus with ophthalmic complications |
| 25010 Dmii Keto Nt St Uncntrld | E10.4 Insulin-dependent diabetes mellitus with neurological complications |
| 25011 Dmi Keto Nt St Uncntrld | E10.5 Insulin-dependent DM with peripheral circulatory complications |
| 25012 Dmii Ketoacd Uncontrold | E10.6 Insulin-dependent DM with other specified complications |
| 25013 Dmi Ketoacd Uncontrold | E10.7 Insulin-dependent diabetes mellitus with multiple complications |
| 25020 Dmii Hprsm Nt St Uncntrl | E10.8 Insulin-dependent diabetes mellitus with unspecified complications |
| 25021 Dmi Hprsm Nt St Uncntrld | E10.9 Insulin-dependent diabetes mellitus without complications |
| 25022 Dmii Hprosmlr Uncontrold | E11.0 Non-insulin-dependent diabetes mellitus with coma |
| 25023 Dmi Hprosmlr Uncontrold | E11.1 Non-insulin-dependent diabetes mellitus with ketoacidosis |
| 25030 Dmii O Cm Nt St Uncntrld | E11.2 Non-insulin-dependent diabetes mellitus with renal complications |
| 25031 Dmi O Cm Nt St Uncntrl | E11.3 Non-insulin-dependent DMwith ophthalmic complications |
| 25032 Dmii Oth Coma Uncontrold | E11.4 Non-insulin-dependent DM with neurological complications |
| 25033 Dmi Oth Coma Uncontrold | E11.5 Non-insulin-dependent DM with peripheral circulatory complications |
| 25040 Dmii Renl Nt St Uncntrld | E11.6 Non-insulin-dependent DM with other specified complications |
| 25041 Dmi Renl Nt St Uncntrld | E11.7 Non-insulin-dependent diabetes mellitus with multiple complications |
| 25042 Dmii Renal Uncntrld | E11.8 Non-insulin-dependent DM with unspecified complications |
| 25043 Dmi Renal Uncntrld | E11.9 Non-insulin-dependent diabetes mellitus without complications |
| 25050 Dmii Ophth Nt St Uncntrl | E13.0 Other specified diabetes mellitus with coma |
| 25051 Dmi Ophth Nt St Uncntrld | E13.1 Other specified diabetes mellitus with ketoacidosis |
| 25052 Dmii Ophth Uncntrld | E13.2 Other specified diabetes mellitus with renal complications |
| 25053 Dmi Ophth Uncntrld | E13.3 Other specified diabetes mellitus with ophthalmic complications |
| 25060 Dmii Neuro Nt St Uncntrl | E13.4 Other specified diabetes mellitus with neurological complications |
| 25061 Dmi Neuro Nt St Uncntrld | E13.5 Other specified DM with peripheral circulatory complications |
| 25062 Dmii Neuro Uncntrld | E13.6 Other specified diabetes mellitus with other specified complications |
| 25063 Dmi Neuro Uncntrld | E13.7 Other specified diabetes mellitus with multiple complications |
| 25070 Dmii Circ Nt St Uncntrld | E13.8 Other specified diabetes mellitus with unspecified complications |
| 25071 Dmi Circ Nt St Uncntrld | E13.9 Other specified diabetes mellitus without complications |
| 25072 Dmii Circ Uncntrld |  |
| 25073 Dmi Circ Uncntrld |  |
| 25080 Dmii Oth Nt St Uncntrld |  |
| 25081 Dmi Oth Nt St Uncntrld |  |
| 25082 Dmii Oth Uncntrld |  |
| 25083 Dmi Oth Uncntrld |  |
| 25090 Dmii Unspf Nt St Uncntrl |  |
| 25091 Dmi Unspf Nt St Uncntrld |  |
| 25092 Dmii Unspf Uncntrld |  |
| 25093 Dmi Unspf Uncntrld |  |
|  |  |
|  | *Diagnosis codes* *for diabetes (continued):* |
|  |  |
|  | E14.0 Unspecified diabetes mellitus with coma |
|  | E14.1 Unspecified diabetes mellitus with ketoacidosis |
|  | E14.2 Unspecified diabetes mellitus with renal complications |
|  | E14.3 Unspecified diabetes mellitus with ophthalmic complications |
|  | E14.4 Unspecified diabetes mellitus with neurological complications |
|  | E14.5 Unspecified DM with peripheral circulatory complications |
|  | E14.6 Unspecified diabetes mellitus with other specified complications |
|  | E14.7 Unspecified diabetes mellitus with multiple complications |
|  | E14.8 Unspecified diabetes mellitus with unspecified complications |
|  | E14.9 Unspecified diabetes mellitus without complications |
|  |  |
|  | |
| Exclude cases: | |
| - transferring from another institution | |
| - pregnancy, childbirth, and puerperium) | |
| - newborn and other neonates) | |
| - with trauma diagnosis code in any field | |
| - same day/day only admissions (admissions with a length of stay less than 24 hours. In those countries where a timestamp of admission or discharge is not available cases with a length of stay of 0 days shall be excluded. | |
|  | |
| Exclude trauma diagnosis codes: | |
|  | |
| **ICD-9-CM** | **ICD-10-WHO** |
| 8950 Amputation Toe | S78.0 Traumatic amputation at hip joint |
| 8951 Amputation Toe-Complicat | S78.1 Traumatic amputation at level between hip and knee |
| 8960 Amputation Foot, Unilat | S78.9 Traumatic amputation of hip and thigh, level unspecified |
| 8961 Amput Foot, Unilat-Compl | S88.0 Traumatic amputation at knee level |
| 8962 Amputation Foot, Bilat | S88.1 Traumatic amputation at level between knee and ankle |
| 8963 Amputat Foot, Bilat-Comp | S88.9 Traumatic amputation of lower leg, level unspecified |
| 8970 Amput Below Knee, Unilat | S98.0 Traumatic amputation of foot at ankle level |
| 8971 Amputat Bk, Unilat-Compl | S98.1 Traumatic amputation of one toe |
| 8972 Amput Above Knee, Unilat | S98.2 Traumatic amputation of two or more toes |
| 8973 Amput Abv Kn, Unil-Compl | S98.3 Traumatic amputation of other parts of foot |
| 8974 Amputat Leg, Unilat Nos | S98.4 Traumatic amputation of foot, level unspecified |
| 8975 Amput Leg, Unil Nos-Comp | T05.3 Traumatic amputation of both feet |
| 8976 Amputation Leg, Bilat | T05.4 Traumatic amputation of 1 foot and other leg [any level, except foot] |
| 8977 Amputat Leg, Bilat-Compl | T05.5 Traumatic amputation of both legs [any level] |
|  | T13.6 Traumatic amputation of lower limb, level unspecified |
|  | |
| **Denominator:** 100,000 Population (age 15+ years). | |
| **Diabetes Short-term Complications Admission Rate** | |
|  | |
| **Numerator:** All non-maternal/non-neonatal hospital discharges (age 15+) with a principal diagnosis code for Diabetes short-term complications (i.e. ketoacidosis, hyperosmolarity, coma) in a specified year. | |
|  | |
| Diabetes short-term diagnostic codes: | |
| **ICD-9-CM** | **ICD-10-WHO** |
| 25010 Dm Keto T2, Dm Cont | E100 Insulin-dependent diabetes mellitus with coma |
| 25011 Dm Keto T1, Dm Cont | E101 Insulin-dependent diabetes mellitus with ketoacidosis |
| 25012 Dm Keto T2, Dm Uncont | E110 Non-insulin-dependent diabetes mellitus with coma |
| 25013 Dm Keto T1, Dm Uncont | E111 Non-insulin-dependent diabetes mellitus with ketoacidosis |
| 25020 Dm W/ Hyprosm T2, Dm Cont | E130 Other specified diabetes mellitus with coma |
| 25021 Dm W/ Hyprosm T1, Dm Cont | E131 Other specified diabetes mellitus with ketoacidosis |
| 25022 Dm W/ Hyprosm T2, Dm Uncnt | E140 Unspecified diabetes mellitus with coma |
| 25023 Dm W/ Hyprosm T1, Dm Uncnt | E141 Unspecified diabetes mellitus with ketoacidosis |
| 25030 Dm Coma Nec Typ Ii, Dm Cnt |  |
| 25031 Dm Coma Nec T1, Dm Cont |  |
| 25032 Dm Coma Nec T2, Dm Uncont |  |
| 25033 Dm Coma Nec T1, Dm Uncont |  |
|  |  |
|  | |
| Exclude cases: | |
| - transferring from another institution | |
| - pregnancy, childbirth, and puerperium) | |
| - newborn and other neonates) | |
| - with trauma diagnosis code in any field | |
| - same day/day only admissions (admissions with a length of stay less than 24 hours. In those countries where a timestamp of admission or discharge is not available cases with a length of stay of 0 days shall be excluded. | |
| **Denominator:** 100,000 Population (age 15+ years). | |

| **CHF admission rate** | |
| --- | --- |
| **Numerator:** All non-maternal/non-neonatal hospital discharges (age 15+) with principal diagnosis code for Congestive Heart Failure (CHF) in a specified year. | |
| CHF diagnostic codes: | |
| **ICD-9-CM** | **ICD-10-WHO** |
| 39891 Rheumatic Heart Failure | I11.0 Hypertensive heart disease with (congestive) heart failure |
| 40201 Mal Hypert Hrt Dis W Chf | I13.0 Hypertensive heart and renal disease with (congestive) heart failure |
| 40211 Benign Hyp Hrt Dis W Chf | I13.2 Hypertensive heart and renal disease with both (congestive) heart failure and renal failure |
| 40291 Hyperten Heart Dis W Chf | I50.0 Congestive heart failure |
| 40401 Mal Hyper Hrt/Ren W Chf | I50.1 Left ventricular failure |
| 40403 Mal Hyp Hrt/Ren W Chf/Rf | I50.9 Heart failure, unspecified |
| 40411 Ben Hyper Hrt/Ren W Chf |  |
| 40413 Ben Hyp Hrt/Ren W Chf/Rf |  |
| 40491 Hyper Hrt/Ren Nos W Chf |  |
| 40493 Hyp Ht/Ren Nos W Chf/Rf |  |
| 4280 Congestive Heart Failure |  |
| 4281 Left Heart Failure |  |
| 42820 Systolic Hrt Failure Nos Oct02- |  |
| 42821 Ac Systolic Hrt Failure Oct02- |  |
| 42822 Chr Systolic Hrt Failure Oct02- |  |
| 42823 Ac On Chr Syst Hrt Fail Oct02- |  |
| 42830 Diastolc Hrt Failure Nos Oct02- |  |
| 42831 Ac Diastolic Hrt Failure Oct02- |  |
| 42832 Chr Diastolic Hrt Fail Oct02- |  |
| 42833 Ac On Chr Diast Hrt Fail Oct02- |  |
| 42840 Syst/Diast Hrt Fail Nos Oct02- |  |
| 42841 Ac Syst/Diastol Hrt Fail Oct02- |  |
| 42842 Chr Syst/Diastl Hrt Fail Oct02- |  |
| 42843 Ac/Chr Syst/Dia Hrt Fail Oct02- |  |
| 4289 Heart Failure Nos |  |
|  |  |
| \| Exclude cases: \| \| --- \| \| - transferring from another institution \| \| - pregnancy, childbirth, and puerperium) \| \| - newborn and other neonates)  - with cardiac procedure codes in any field \| \| - with trauma diagnosis code in any field \| \| - same day/day only admissions (admissions with a length of stay less than 24 hours. In those countries where a timestamp of admission or discharge is not available cases with a length of stay of 0 days shall be excluded. \| | |
|  | |
| Denominator: 100,000 Population (age 15+ years). | |

| **Hypertension Admission Rate** | | |
| --- | --- | --- |
| **Numerator:** All non-maternal hospital discharges (age 15+) with principal diagnosis code for Hypertension in a specified year. | | |
|  | | |
| Hypertension diagnostic codes: | | |
|  | | |
| **ICD-9-CM** | | **ICD-10-WHO** |
| 4010 Malignant Hypertension | | I10 Essential (primary) hypertension |
| 4019 Hypertension Nos | | I119 Hypertensive heart disease without (congestive) heart failure |
| 40200 Mal Hyperten Hrt Dis Nos | | I129 Hypertensive renal disease without renal failure |
| 40210 Ben Hyperten Hrt Dis Nos | | I139 Hypertensive heart and renal disease, unspecified |
| 40290 Hypertensive Hrt Dis Nos | |  |
| 40300 Mal Hyp Ren W/O Ren Fail | |  |
| 40310 Ben Hyp Ren W/O Ren Fail | |  |
| 40390 Hyp Ren Nos W/O Ren Fail | |  |
| 40400 Mal Hy Ht/Ren W/O Chf/Rf | |  |
| 40410 Ben Hy Ht/Ren W/O Chf/Rf | |  |
| 40490 Hy Ht/Ren Nos W/O Chf/Rf | |  |
|  | | |
| Exclude cases: | | |
| - transferring from another institution | | |
| - pregnancy, childbirth, and puerperium) | | |
| - newborn and other neonates)  - with cardiac procedure codes in any field | | |
| - with cardiac procedure codes in any field | | |
| - same day/day only admissions (admissions with a length of stay less than 24 hours. In those countries where a timestamp of admission or discharge is not available cases with a length of stay of 0 days shall be excluded. | | |
|  | | |
|  | | |
|  | | |
| **Denominator:** 100,000 Population (age 15+ years). | | |
|  | |  |
|  | |  |
